# Associations Between Genotype and Peripheral Complement Proteins in First-Episode Psychosis: Evidences From C3 and C4

**DOI:** 10.3389/fgene.2021.647246

**Published:** 2021-07-09

**Authors:** Yu Chen, Zhenguo Zhao, Fen Lin, Lifang Wang, Zheng Lin, Weihua Yue

**Affiliations:** ^1^Institute of Mental Health, Peking University Sixth Hospital, Beijing, China; ^2^Key Laboratory of Mental Health, National Clinical Research Center for Mental Disorders, Chinese Academy of Medical Sciences Research Unit (No. 2018RU006), Ministry of Health (Peking University), Beijing, China; ^3^Second Hospital Zhejiang University School of Medicine (SAHZU), Hangzhou, China; ^4^PKU-IDG/McGovern Institute for Brain Research, Peking University, Beijing, China

**Keywords:** first-episode psychosis, complement protein concentration, single nucleotide polymorphism, Chinese Han population, case-control studies

## Abstract

Schizophrenia is a common neuropsychiatric disorder with complex pathophysiology. Recent reports suggested that complement system alterations contributed to pathological synapse elimination that was associated with psychiatric symptoms in schizophrenia. Complement component 3 (C3) and complement component 4 (C4) play central roles in complement cascades. In this study, we compared peripheral C3 and C4 protein levels between first-episode psychosis (FEP) and healthy control (HC). Then we explored whether single nucleotide polymorphisms (SNPs) at C3 or C4 genes affect peripheral C3 or C4 protein levels. In total, 181 FEPs and 204 HCs were recruited after providing written informed consent. We measured serum C3 and C4 protein levels using turbidimetric inhibition immunoassay and genotyped C3 and C4 polymorphisms using the Sequenom MassArray genotyping. Our results showed that three SNPs were nominally associated with schizophrenia (rs11569562/C3: A > G, *p* = 0.048; rs2277983/C3: A > G, *p* = 0.040; rs149898426/C4: G > A, *p* = 0.012); one haplotype was nominally associated with schizophrenia, constructed by rs11569562–rs2277983–rs1389623 (GGG, *p* = 0.048); FEP had higher serum C3 and C4 (both *p* < 0.001) levels than HC; rs1389623 polymorphisms were associated with elevated C3 levels in our meta-analysis (standard mean difference, 0.50; 95% confidence interval, 0.30 to 0.71); the FEP with CG genotype of rs149898426 had higher C4 levels than that with GG genotypes (*p* = 0.005). Overall, these findings indicated that complement system altered in FEP and rs149898426 of C4 gene represented a genetic risk marker for schizophrenia likely through mediating complement system. Further studies with larger sample sizes needs to be validated.

## Highlights

- Peripheral C3 and C4 protein levels were increased in first-episode psychosis (FEP).
- The rs149898426 polymorphisms of C4 gene were associated with schizophrenia.
- The CG genotype of rs149898426 were associated with higher serum C4 level in FEP comparing with GG genotype.

## Introduction

Schizophrenia is a severe and complicated neuropsychiatric disorder characterized by hallucinations, delusions and cognitive dysfunction (Kahn and Keefe, 2013; Kahn et al., 2015; Owen et al., 2016). While many studies attributed the causes of schizophrenia to biological or environmental factors, the etiology of schizophrenia are still unclear. Several lines of evidence suggested that dysregulation of the immune system contributed to the development of schizophrenia. Epidemiological studies found that infection and autoimmune disorders were associated with schizophrenia (Brown and Derkits, 2010; Benros et al., 2011, 2014; Arias et al., 2012; Khandaker et al., 2013). Besides, many cross-sectional studies reported that proinflammatory cytokines and C-reactive protein (CRP) were increased in schizophrenia compared with healthy control (HC) (Miller et al., 2011; Fineberg and Ellman, 2013; Fernandes et al., 2016). Lastly, antipsychotics has been demonstrated to have immunomodulatory effects, for example, clozapine (Hinze-Selch et al., 1998; Roge et al., 2012). Thus, immune dysregulation could represent a vulnerability factor for schizophrenia.

Complement system, an important part of the innate immune system, play important roles in clearing microbes and damaged cells from an organism, triggering inflammation and destroying foreign invaders (Merle et al., 2015a,b). Recent evidence identified that complement system orchestrated the balance for neurodevelopmental processes (Stephan et al., 2012; Ricklin et al., 2016). For example, overactivation of complement system contributed to neurotoxicity and pathological synapse loss through microglia, leading to progressive dysfunction in Alzheimer disease (AD) (Shi et al., 2017; Heneka et al., 2018).

Complement component 3 (C3) and complement component 4 (C4) play dominant roles in complement cascades. The C3 gene is located on chromosome 19, consisting of 41 exons and the mRNA has 5101 bp. C3 is a convergent point for activation both classical and alternative complement pathways (Merle et al., 2015a,b). Genetic evidence suggests that the polymorphisms of C3 were associated with risk of schizophrenia (Rudduck et al., 1985; Fañanás et al., 1992; Ni et al., 2015; Zhang et al., 2018). And patients with schizophrenia had increased C3 protein levels in peripheral blood (Santos Sória et al., 2012; Ali et al., 2017) and serum C3 concentrations were positively correlated with PANSS scores (Li et al., 2016). The C4 gene has two isoforms, C4A and C4B, located within the major histocompatibility complex (MHC) region on chromosome 6. C4A was proved to increase the risk for schizophrenia through mediating synapse pruning during postnatal development (Sekar et al., 2016).

The elucidation of pathophysiology in the early stages of schizophrenia may help understand disease etiology. First-episode psychosis (FEP) refers to the first time a person outwardly experiences symptoms of psychosis. Although some symptoms were unspecific in FEP, the underlying biological processes has changed, especially upregulating inflammatory status. Given that C3 and C4 are major plasma proteins of the complement pathway and are widely measured parameters during clinical practice, we investigated whether C3 or C4 protein levels are different between FEP and HC from our clinical data. Considering previous reports that C3 or C4 gene has multiple single nucleotide polymorphisms (SNPs) that were at the genome-wide level (Yang et al., 2012), we selected several SNPs at C3 (rs11569562, rs2277983, rs1389623) and C4 (rs406658, rs2746414, rs149898426) genes to explore the associations between genotype and complement protein concentration in FEP.

## Materials and Methods

### Subjects

A total of 181 FEPs (94 males, 87 females; mean age: 29.9 ± 10.0 years) and 204 HCs (96 males, 108 females; mean age: 30.0 ± 10.2 years) were recruited from Peking University Sixth Hospital. The diagnosis was made according to the fourth edition of the Diagnostic and Statistical Manual of Mental Disorders (DSM-IV) criteria using a Structured Clinical Interview and people with FEP did not take medication regularly. We excluded HC with a history of mental health or neurological diseases from communities through simple unstructured interviews conducted by psychiatrists. All participants were given written informed consent before the study began. The study was conducted under established ethical standards and was approved by the ethics committee of Peking University Sixth Hospital.

### SNP Selection and Genotyping

We collected 5 mL venous blood from each subject, separated out serum by centrifugation, and stored it at –80°C until laboratory analysis. The genomic DNA was extracted from using the QIAamp DNA Blood Mini Kit. We selected 3 haplotype-tagging single nucleotide polymorphisms (tagSNPs) by using the UCSC (GRCh37/hg19) genome browser^1^ for C3 and C4, respectively (C3: rs11569562, rs2277983, rs1389623; C4: rs406658, rs2746414, rs149898426). Genotyping was conducted for 6 tagSNPs by using the platform of the Sequenom MassArray system (Sequenom, San Diego, CA, United States). Locus-specific polymerase chain reaction (PCR) and detection primers were designed with the MassARRAY Assay Design Version 3.0 software (Sequenom, San Diego, CA, United States). The DNA samples were amplified by multiplex PCR reactions and the obtained products were used for locus-specific single-base extension reaction. The final products were desalted and transferred to 384-element SpectroCHIP arrays. Allele typing was performed using MALDI-TOF MS spectroscopy. The mass spectrum was analyzed by the MassARRAY TYPER software (Sequenom, San Diego, CA, United States). All DNA samples were analyzed in technical duplicates. We genotyped each sample thrice to minimize genotyping errors and only consensus genotypes were processed for further analysis. Genotyping primer sequences are indicated in Table 1.

**TABLE 1:** SNP information and PCR and extension primer sequences from Sequenom SNP genotyping.

SNP	Position	Location	Second-PCRP	First-PCRP	UEP_SEQ
rs11569562	6678742	Intron	ACGTTGGATGTACTATGCAATGAGAGTGAC	ACGTTGGATGTGACTGTAGTTTTCCGTGGC	CCATGAGGCTACAGTATT
rs2277983	6679552	Intron	ACGTTGGATGGAAGATGAGAGGATAAGGGC	ACGTTGGATGCAGGTCTGAAGACTGAGAAC	CTCCCTCCAAAGACCA
rs1389623	6684186	Intron	ACGTTGGATGATGATTGTGTACTTTCCTCC	ACGTTGGATGTGTGAGACCACAGCAATGAC	AGCAATGACCACGTAAG
rs406658	32028747	Coding sequence	ACGTTGGATGTTGAAGGTCCTGAGTTTGGC	ACGTTGGATGGGACAGAAGCCAGTTAGATG	CAGTTTCTCAGGCGA
rs2746414	32029189	Coding sequence	ACGTTGGATGGTTTTCTTCCAGGAAGCCTC	ACGTTGGATGAGCCCAGCACTTGCTTTCTC	TCCATCTCAAAGGCAA
rs149898426	32029352	intron	ACGTTGGATGCAACAACCTCATGGCAATGG	ACGTTGGATGCACTCAGGGATCCTAAGGTC	GCTCACTGCCAGAGC

### Measurement of Complement Protein

Serum C3 and C4 levels were measured using the turbidimetric inhibition immunoassay in an automatic chemistry analyzer, ROCHE (Roche Molecular Systems, Inc., Basel, Switzerland) module Cobas 8000 (C702). The assays were performed strictly by the manufacturer’s recommended protocol. All samples were analyzed in duplicates and the mean of the two measurements was used in the analysis.

### Statistical Analyses

Pairwise linkage disequilibrium (LD), haplotype construction, and genetic association analysis were performed by using Haploview 4.2 software^2^. Deviation of the genotype and allele frequency from the Hardy–Weinberg equilibrium were analyzed using a chi-square goodness-of-fit test. We conducted *t*-tests and a univariate analysis of covariance (ANCOVA) to examine the difference of serum complement protein levels (C3 and C4) between FEP and HC, considering that age and sex may affect serum complement protein levels. A two-way analysis of variance (ANOVA) was used to explore the interaction effects of group and genotype. *Post hoc* analyses were performed using a Tukey test. When the result from *F*-test was marginal significant (0.05 < *p* < 0.1), we would conduct a meta-analysis of the SNP for case group and control group to increase the statistical power. Statistical analyses were performed using R statistical software^3^. Data were expressed as the mean ± standard deviation (SD) or as number (proportion). Results were considered nominally significant at *p* < 0.05 (two-tailed). Multiple testing adjustment will be controlled via Bonferroni adjustment for comparison of genotype and allele distribution for C3 and C4 gene. *P* < 0.05/3 was considered as significant for the reason that the total number of independence tests (excluding LD) was 3 for all 6 SNPs across two genes.

## Results

### Single Polymorphism Analysis

We observed no association of rs11569562, rs1389623, s406658, and rs2746414 polymorphism with schizophrenia for genotypes and alleles. There were two nominally significant associations between rs11569562 (*p* = 0.048) and rs2277983 (*p* = 0.040) alleles distributions and schizophrenia but no association between genotypes and schizophrenia. The genotypes (*p* = 0.0205) and alleles (*p* = 0.012 < 0.05/3) of rs149898426 were significantly different between FEP and HC. The genotypes and alleles frequencies of each polymorphism were presented in Table 2.

**TABLE 2:** Comparison of genotype and allele distribution of 6 SNPs of C3 and C4 gene between FEP and HC.

Makers/gene	Genotype *N* (Freq.)				Chi-square (df = 2)	*p*-Value	HWE *P*	Allele *N* (freq.)		Chi-square (df = 1)	*p*-Value	OR (95% CI)
rs11569562/C3	A/A	A/G	G/G	NA				A	G			
FEP	54 (0.298)	89 (0.492)	38 (0.210)		4.325	0.114	0.905	197 (0.544)	165 (0.456)	3.886	0.048^*a*^	0.751 (0.566–0.999)
HC	43 (0.211)	107 (0.525)	54 (0.265)				0.457	193 (0.473)	215 (0.527)			
rs2277983/C3	A/A	A/G	G/G					A	G			
FEP	54 (0.298)	90 (0.497)	37 (0.204)		4.484	0.106	0.964	198 (0.547)	164 (0.453)	4.199	0.040^*a*^	0.743 (0.559–0.987)
HC	43 (0.212)	106 (0.522)	54 (0.266)	1			0.499	192 (0.473)	214 (0.527)			
rs1389623/C3	A/A	A/G	G/G					A	G			
FEP	3 (0.017)	41 (0.227)	137 (0.757)		0.178	0.915	0.973	47 (0.130)	315 (0.870)	0.040	0.841	1.044 (0.683–1.596)
HC	4 (0.020)	43 (0.211)	157 (0.767)				0.603	51 (0.125)	357 (0.875)			
rs406658/C4	A/A	A/C	C/C					A	C			
FEP	1 (0.006)	33 (0.189)	141 (0.806)	6	1.939	0.379	0.529	35 (0.100)	315 (0.900)	1.480	0.224	0.736 (0.468–1.158)
HC	3 (0.015)	47 (0.233)	152 (0.752)	2			0.769	53 (0.131)	351 (0.869)			
rs2746414/C4	A/A	A/G	G/G					A	G			
FEP	0 (0)	29 (0.160)	152 (0.840)		1.977	0.372	0.241	29 (0.080)	333 (0.920)	0.479	0.489	0.811 (0.490–1.341)
HC	2 (0.001)	35 (0.174)	164 (0.816)	3			0.931	39 (0.097)	363 (0.903)			
rs149898426/C4	C/C	C/G	G/G					C	G			
FEP	0 (0)	28 (0.156)	152 (0.844)	1	7.775	0.021^*a*^	0.258	28 (0.078)	332 (0.922)	6.344	0.012^*b*^	0.527 (0.325–0.853)
HC	3 (0.016)	47 (0.245)	142 (0.740)	12			0.690	53 (0.138)	331 (0.862)			

*FEP, first-episode psychosis; HC, healthy control; Freq., frequencies; HWE P, The *p*-value for Hardy-Weinberg equilibrium tests; OR, odds ratio; CI, confidence interval;*

*NA indicates a missing value.*

*^*a*^Denotes a significant association (*P* < 0.05).*

*^*b*^Denotes a significant association after Bonferroni adjustment (*P* < 0.05/3 = 0.017).*

### Haplotype-Based Association Analysis

**FIGURE 1:**
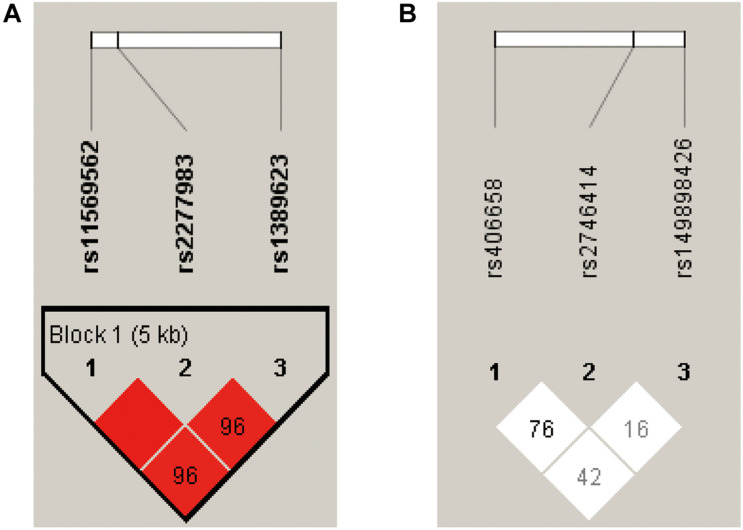
The pairwise linkage disequilibrium (LD) analysis was applied to detect the inter-marker relationship of the three SNPs at the C3 and C4 gene receptively, by using the *D*’ values. According to the *D*’ values in this figure, **(A)** three SNPs constructed into one haplotype block in C3 gene. **(B)** No haplotype block was detected in C4 gene.

The LD map and block structures of C3 and C4 polymorphisms were shown in Figure 1. A haplotype block of C3 was constructed by rs11569562, rs2277983, and rs1389623: GGG haplotype was significantly associated with schizophrenia (χ^2^ = 3.922, *p* = 0.048); while after 10,000 times of permutation tests, the difference of haplotype GGG frequencies between FEP and HC were not significant (empirical *p*-value was 0.104) (see details in Table 3 and Figure 1A). No haplotype blocks were found in C4 gene (Figure 1B).

**TABLE 3:** Comparison of haplotype frequencies at the C3 gene between FEP and HC.

Haplotypes	Freq. case	Freq. control	Chi-square	*p*-Value	Empirical *p*-Value
Block (rs11569562–rs2277983–rs1389623)					
GGG	0.524	0.452	3.922	0.048	0.104
AAG	0.351	0.415	3.320	0.069	0.158
AAA	0.125	0.129	0.092	0.761	1.000

*FEP, first-episode psychosis; HC, healthy control; Freq., frequency. Haplotypes with frequency < 0.01 in FEP and HC were dropped. Empirical *p*-value: the *p*-value of the permutation tests (number of iterations, 10,000).*

### Complement Protein Levels

**FIGURE 2:**
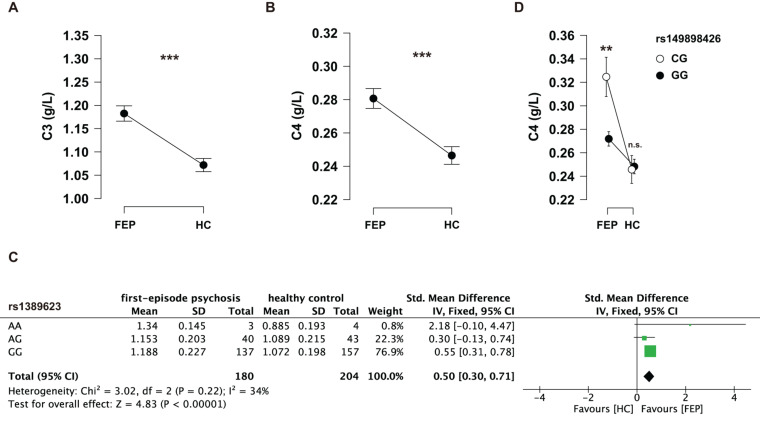
**(A)** Serum C3 protein levels between FEP and HC. **(B)** Serum C4 protein levels between FEP and HC. Data were analyzed by Student’s *t*-tests. **(C)** Serum C4 levels among the genotypes of rs149898426 (CG and GG) in FEP and HC receptively. Data were analyzed by a two-way ANOVA with Tukey *post hoc* tests. Data shown in the figure represent mean ± SEM. **P < 0.01; ***P < 0.001; n.s.: non-significant. **(D)** A meta-analysis of the genotypes of rs1389623 on serum C3 levels.

One sample testing C3 protein levels was excluded for being three SDs above the mean. The serum C3 protein levels (mean ± SD) in cases and controls were 1.18 ± 0.22 and 1.07 ± 0.20 g/L; the serum C4 protein levels (mean ± SD) in cases and controls were 0.28 ± 0.08 and 0.25 ± 0.08 g/L, respectively. *T*-tests showed that FEP had higher serum C3 (*t* = 5.11, *p* < 0.001, Cohen’s *d* = 0.52) and C4 (*t* = 4.21, *p* < 0.001, Cohen’s *d* = 0.44) protein levels than HC (Figures 2A,B). Although the differences of serum C3 and C4 protein levels were not significant considering age and sex in cases and controls, respectively (see details in Table 4), age- and sex-adjusted comparisons were performed with ANCOVA: the differences of serum C3 (*F* = 26.55, *p* < 0.001, η^2^ = 0.065) and C4 (*F* = 18.27, *p* < 0.001, η^2^ = 0.046) protein levels remained significant.

**TABLE 4:** Subgroups of serum C3 and C4 levels according to sex and clinical information.

	Group	Sex	N	Mean ± SD (g/L)	*P*
C3	FEP	Male	94	1.16 ± 0.24	0.44
		Female	86	1.20 ± 0.20	
	HC	Male	96	1.07 ± 0.22	0.77
		Female	108	1.08 ± 0.19	
C4	FEP	Male	94	0.28 ± 0.09	0.45
		Female	87	0.27 ± 0.07	
	HC	Male	96	0.24 ± 0.08	0.68
		Female	108	0.24 ± 0.07	

*FEP, first-episode psychosis; HC, healthy control. One sample information (C3 for Female in cases) was lost for technical errors.*

### SNP-Group Interactions in Complement Proteins

Serum C3 and C4 protein levels of individual genotypes were evaluated between the case and control groups using two-way ANOVAs. We found that the interaction effects of group (FEP vs. HC) and rs1389623 were marginal significant (*F* = 2.81, *p* = 0.062, partial η^2^ = 0.015) on serum C3 levels (see Supplementary Tables 1–2). A meta-analysis showed that rs1389623 polymorphism were associated with elevated serum C3 levels (*Z* = 4.83, *p* < 0.001) (see details in Figure 2C). There was no interaction effect between other genotypes and group on serum C3 protein levels (see Supplementary Tables 3–6). Besides, we excluded all CC genotypes in a two-way ANOVAs (group: FEP, HC × rs149898426 polymorphisms: CG, GG) on C4 protein levels, for the reason that there were three subjects with CC genotypes in HC and no subject in FEP. The interaction effects of group and rs149898426 were significant (*F* = 7.33, *p* = 0.007, partial η^2^ = 0.02) (see Supplementary Tables 7–8). *Post hoc* tests found that FEP who carry the CG genotypes of rs149898426 had higher serum C4 protein levels than the patients who carry the GG genotypes (*t* = 3.34, *p*_*tukey*_ = 0.005) while HC not (*t* = −0.198, *p*_*tukey*_ = 0.997) (Figure 2D). No other interaction of genotypes and group was significant in serum C4 protein levels (see Supplementary Tables 9–12).

## Discussion

The present study confirmed that complement system overactivated in FEP, which were correlated with genetic factors. We observed that FEP had higher serum C3 and C4 protein levels than HC, suggesting that aberrant expression of complement proteins may be potential biomarkers for schizophrenia; C4 polymorphism affected serum C4 protein levels in FEP but not in HC, suggesting that genotype may be considered as an important risk factor for the development of schizophrenia.

The first finding was that FEP had increased serum C3 and C4 protein levels compared with HC. The results replicated many previous findings in schizophrenia (Maes et al., 1997) and extended to patients that were not FEP. But two other studies had no similar findings in FEP (Kopczynska et al., 2019; Laskaris et al., 2019). One possible reason is that the sample size may not be sufficient to reach the statistical differences; both studies had lower sample sizes than our study. Another potential cause for this divergence of outcomes is race or ethnicity. In our study, study participants are limited to the Chinese Han population, while various racial groups were involved in other studies. However, one study pointed that individuals at ultra-high risk (UHR) for psychosis had elevated C3 and C4 protein levels (Laskaris et al., 2019), suggesting that alteration of complement system accompanied the development of schizophrenia. These results should be considered exploratory and further studies with larger cohorts would be required to confirm results.

To our knowledge, this is the first survey that compares serum C3 and C4 levels with their SNP in FEP of the China Han population. Two recent papers investigated the roles of C3 polymorphism in susceptibility to schizophrenia in the China Han population (Ni et al., 2015; Zhang et al., 2018). Although SNP rs11569562 in C3 was not associated with schizophrenia in the Chinese Han population (Zhang et al., 2018) consisting of 1086 patients with schizophrenia and 1154 HCs, patients with all genotypes of rs11569562 have higher serum C3 levels than the controls similar to our study. Other studies using expression quantitative trait locus (eQTL) analysis revealed that rs2277984, which is adjacent to rs2277983 in our study, regulates C3 expression in the liver (Zhang et al., 2017), suggesting that SNPs in the C3 gene affected the expression of C3 genes to further impact the C3 protein translation. Similarly, one study found that in the region near C4 of chromosome 6, the more strongly an SNP associated with schizophrenia, the more strongly it correlated with predicted C4A expression (Sekar et al., 2016). In short, the genotype of the complement gene had effects on its expression, which concurs with our results.

The present study has a major limitation that the sample size was small and may underpower the whole study in terms of C3 and C4 genotype distribution. Therefore, our results should be interpreted with caution. Further studies with larger sample sizes will be required to achieve sufficient statistical power and elucidate a potential link between complement system and schizophrenia in China Han population.

## Conclusion

Our results showed that first-episode psychosis had higher serum C3 and C4 protein levels than healthy control. C4 gene polymorphisms affected C4 protein levels in peripheral blood: the FEP carrying CG genotypes of rs149898426 had higher serum C4 protein levels than that of GG genotypes. The available evidence that complement proteins were elevated in FEP and polymorphisms of complement genes played a contributing role in regulating peripheral complement proteins concentration.

## Data Availability Statement

The data presented in the study are deposited in the Peking University Open Research Data repository, accession number 10635 (http://opendata.pku.edu.cn/api/access/datafile/10635).

## Ethics Statement

The studies involving human participants were reviewed and approved by the Ethics Committee of Peking University Sixth Hospital. The patients/participants provided their written informed consent to participate in this study.

## Author Contributions

WY provided the funds and designed the study. YC analyzed the data and wrote the draft of the manuscript. ZZ recruited schizophrenia patients and collected peripheral blood samples. Primers were designed by FL and LW. ZL supervised this study. All authors contributed to the article and approved the submitted version.

## Conflict of Interest

The authors declare that the research was conducted in the absence of any commercial or financial relationships that could be construed as a potential conflict of interest. The reviewer QW declared a past co-authorship with the authors LW and WY to the handling editor.

## Footnotes

1 http://genome.ucsc.edu/

2 http://www.broadinstitute.org/haploview/

3 https://www.r-project.org
