# Supplementary material for: Associations Between Genotype and Peripheral Complement Proteins in First-Episode Psychosis: Evidences From C3 and C4
Source: Front Genet. 2021 Jul 9;12:647246. doi: 10.3389/fgene.2021.647246 (PMC8301372; doi:10.3389/fgene.2021.647246)
Supplement: Supplementary file 1 [file Data_Sheet_1.docx]

**Supplementary Material**

**1.Effects of rs1389623 polymorphism on serum C3 protein levels between first-episode psychosis and healthy controls**

Tables S1 and S2 showed that rs1389623 polymorphism did not affect serum C3 protein levels (p = 0.929) and the interaction effects were marginal significant (p = 0.062).

| **Table S1 A two-way ANOVA test regarding rs1389623 for serum C3 protein levels (g/L) between first-episode psychosis and healthy controls** | | | | | | | | | | | | | |
| --- | --- | --- | --- | --- | --- | --- | --- | --- | --- | --- | --- | --- | --- |
| **Cases** | | **Sum of Squares** | | **df** | | **Mean Square** | | **F** | | **p** | | **η²_p_** | |
| group |  | 0.624 |  | 1 |  | 0.624 |  | 13.986 |  | < .001 |  | 0.036 |  |
| rs1389623 |  | 0.007 |  | 2 |  | 0.003 |  | 0.074 |  | 0.929 |  | 3.900e -4 |  |
| group * rs1389623 |  | 0.250 |  | 2 |  | 0.125 |  | 2.805 |  | 0.062 |  | 0.015 |  |
| Residuals |  | 16.876 |  | 378 |  | 0.045 |  |  |  |  |  |  |  |

**Table S2 Descriptives regarding rs1389623 for serum C3 protein levels (g/L) between first-episode psychosis and healthy control**

| **group** | | **rs1389623** | | **Mean** | | **SD** | | **N** | |
| --- | --- | --- | --- | --- | --- | --- | --- | --- | --- |
| FEP |  | AA |  | 1.340 |  | 0.145 |  | 3 |  |
|  |  | AG |  | 1.153 |  | 0.203 |  | 40 |  |
|  |  | GG |  | 1.188 |  | 0.227 |  | 137 |  |
| HC |  | AA |  | 0.885 |  | 0.193 |  | 4 |  |
|  |  | AG |  | 1.089 |  | 0.215 |  | 43 |  |
|  |  | GG |  | 1.072 |  | 0.198 |  | 157 |  |

FEP: first-episode psychosis; HC: healthy control.

**2.Effects of rs11569562 polymorphism on s****erum C3 protein levels between first-episode psychosis and healthy controls**

Tables S3 and S4 showed that rs11569562 polymorphism did not affect serum C3 protein levels (p = 0.203) and the interaction effects were not significant (p = 0.131).

**Table S3 A two-way ANOVA test regarding rs11569562 for serum C3 protein levels (g/L) between first-episode psychosis and healthy controls**

| **Cases** | | **Sum of Squares** | | **df** | | **Mean Square** | | **F** | | **p** | | **η²_p_** | |
| --- | --- | --- | --- | --- | --- | --- | --- | --- | --- | --- | --- | --- | --- |
| group |  | 1.330 |  | 1 |  | 1.330 |  | 29.853 |  | <.001 |  | 0.073 |  |
| rs11569562 |  | 0.143 |  | 2 |  | 0.071 |  | 1.603 |  | 0.203 |  | 0.008 |  |
| group * rs11569562 |  | 0.182 |  | 2 |  | 0.091 |  | 2.041 |  | 0.131 |  | 0.011 |  |
| Residuals |  | 16.839 |  | 378 |  | 0.045 |  |  |  |  |  |  |  |

**Table S4 Descriptives regarding rs11569562 for serum C3 protein levels (g/L) between first-episode psychosis and healthy control**

| **group** | **rs11569562** | **Mean** | | | **SD** | **N** | |  |
| --- | --- | --- | --- | --- | --- | --- | --- | --- |
| FEP  HC | AA |  | 1.216 |  | 0.196 |  | 53 |  |
|  | AG |  | 1.142 |  | 0.221 |  | 89 |  |
|  | GG |  | 1.231 |  | 0.244 |  | 38 |  |
|  | AA |  | 1.060 |  | 0.217 |  | 43 |  |
|  | AG |  | 1.074 |  | 0.202 |  | 107 |  |
|  | GG |  | 1.077 |  | 0.196 |  | 54 |  |

FEP: first-episode psychosis; HC: healthy control.

**3.Effects of rs2277983 polymorphism on serum C3 protein levels between first-episode psychosis and healthy controls**

Tables S5 and S6 showed that rs2277983 polymorphism did not affect serum C3 protein levels (p = 0.248) and the interaction effects were not significant (p = 0.155).

| **Table S5 A two-way ANOVA test regarding rs2277983 for serum C3 protein levels (g/L) between first-episode psychosis and healthy controls** | | | | | | | | | | | | | |
| --- | --- | --- | --- | --- | --- | --- | --- | --- | --- | --- | --- | --- | --- |
| **Cases** | | **Sum of Squares** | | **df** | | **Mean Square** | | **F** | | **p** | | **η²_p_** | |
| group |  | 1.312 |  | 1 |  | 1.312 |  | 29.334 |  | < .001 |  | 0.072 |  |
| rs2277983 |  | 0.125 |  | 2 |  | 0.063 |  | 1.398 |  | 0.248 |  | 0.007 |  |
| group * rs2277983 |  | 0.168 |  | 2 |  | 0.084 |  | 1.875 |  | 0.155 |  | 0.010 |  |
| Residuals |  | 16.866 |  | 377 |  | 0.045 |  |  |  |  |  |  |  |

**Table S6 Descriptives regarding rs2277983 for serum C3 protein levels (g/L) between first-episode psychosis and healthy control**

| **group** | | **rs2277983** | | **Mean** | | **SD** | | **N** | |
| --- | --- | --- | --- | --- | --- | --- | --- | --- | --- |
| FEP |  | AA |  | 1.216 |  | 0.196 |  | 53 |  |
|  |  | AG |  | 1.144 |  | 0.221 |  | 90 |  |
|  |  | GG |  | 1.227 |  | 0.247 |  | 37 |  |
| HC |  | AA |  | 1.060 |  | 0.217 |  | 43 |  |
|  |  | AG |  | 1.075 |  | 0.202 |  | 106 |  |
|  |  | GG |  | 1.077 |  | 0.196 |  | 54 |  |

FEP: first-episode psychosis; HC: healthy control.

**4.Effects of rs149898426 polymorphism on serum C4 protein levels between first-episode psychosis and healthy controls**

For the reason that subjects with CC genotypes was low in each group (FEP: 0 subject; HC: 3 subjects), we excluded subjects with CC genotypes in the two-way ANOVA test. Tables S7 and S8 showed that rs149898426 polymorphism affect serum C4 protein levels (p = 0.015) and the interaction effects were significant (p = 0.007).

**Table S7 A two-way ANOVA test regarding rs149898426 for serum C4 protein levels (g/L) between first-episode psychosis and healthy controls**

| **Cases** | | **Sum of Squares** | | **df** | | **Mean Square** | | **F** | | **p** | | **η²_p_** | |
| --- | --- | --- | --- | --- | --- | --- | --- | --- | --- | --- | --- | --- | --- |
| group |  | 0.149 |  | 1 |  | 0.149 |  | 25.081 |  | < .001 |  | 0.064 |  |
| rs149898426 |  | 0.036 |  | 1 |  | 0.036 |  | 6.033 |  | 0.015 |  | 0.016 |  |
| group * rs149898426 |  | 0.043 |  | 1 |  | 0.043 |  | 7.328 |  | 0.007 |  | 0.020 |  |
| Residuals |  | 2.162 |  | 365 |  | 0.006 |  |  |  |  |  |  |  |

**Table S8 Descriptives regarding rs149898426 for serum C4 protein levels (g/L) between first-episode psychosis and healthy control**

| **group** | | **rs149898426** | | **Mean** | | **SD** | | **N** | |
| --- | --- | --- | --- | --- | --- | --- | --- | --- | --- |
| FEP |  | CG |  | 0.325 |  | 0.088 |  | 28 |  |
|  |  | GG |  | 0.272 |  | 0.076 |  | 152 |  |
| HC |  | CG |  | 0.246 |  | 0.081 |  | 47 |  |
|  |  | GG |  | 0.248 |  | 0.074 |  | 142 |  |

FEP: first-episode psychosis; HC: healthy control.

**5.Effects of rs406658 polymorphism on serum C4 protein levels between first-episode psychosis and healthy controls**

For the reason that subjects with AA genotypes was low in each group (FEP: 1 subject; HC: 3 subjects), we excluded subjects with AA genotypes in the two-way ANOVA test. Tables S9 and S10 showed that rs406658 polymorphism did not affect serum C4 protein levels (p = 0.579) and the interaction effects were marginal significant (p = 0.417).

**Table S9 A two-way ANOVA test regarding rs406658 for serum C4 protein levels (g/L) between first-episode psychosis and healthy controls**

| **Cases** | | **Sum of Squares** | | | | **df** | | | | **Mean Square** | | | | **F** | | | | **p** | | | | **η²_p_** | | | |  |
| --- | --- | --- | --- | --- | --- | --- | --- | --- | --- | --- | --- | --- | --- | --- | --- | --- | --- | --- | --- | --- | --- | --- | --- | --- | --- | --- |
| group |  | 0.049 | |  | | 1 | |  | | 0.049 | |  | | 8.147 | |  | | 0.005 | |  | | 0.022 | |  | |  |
| rs406658 |  | 0.002 | |  | | 1 | |  | | 0.002 | |  | | 0.308 | |  | | 0.579 | |  | | 8.331e-4 | |  | |  |
| group * rs406658 |  | | 0.004 | |  | | 1 | |  | | 0.004 | |  | | 0.660 | |  | | 0.417 | |  | | 0.002 | |  | |
| Residuals |  | 2.231 | |  | | 369 | |  | | 0.006 | |  | |  | |  | |  | |  | |  | |  | |  |

**Table S10 Descriptives regarding rs406658 for serum C4 protein levels (g/L) between first-episode psychosis and healthy control**

| **group** | | **rs406658** | | **Mean** | | **SD** | | **N** | |
| --- | --- | --- | --- | --- | --- | --- | --- | --- | --- |
| FEP |  | AC |  | 0.279 |  | 0.079 |  | 33 |  |
|  |  | CC |  | 0.281 |  | 0.081 |  | 141 |  |
| HC |  | AC |  | 0.259 |  | 0.078 |  | 47 |  |
|  |  | CC |  | 0.245 |  | 0.075 |  | 152 |  |

FEP: first-episode psychosis; HC: healthy control.

**6.Effects of rs2746414 polymorphism on serum C4 protein levels between first-episode psychosis and healthy controls**

For the reason that subjects with AA genotypes was low in each group (FEP: 0 subject; HC: 2 subjects), we excluded subjects with AA genotypes in the two-way ANOVA test. Tables S11 and S12 showed that rs2746414 polymorphism did not affect serum C4 protein levels (p = 0.557) and the interaction effects were not significant (p = 0.776).

**Table S11 A two-way ANOVA test regarding rs2746414 for serum C4 protein levels (g/L) between first-episode psychosis and healthy controls**

| **Cases** | | **Sum of Squares** | | **df** | | **Mean Square** | | **F** | | **p** | | **η² _p_** | |
| --- | --- | --- | --- | --- | --- | --- | --- | --- | --- | --- | --- | --- | --- |
| group |  | 0.053 |  | 1 |  | 0.053 |  | 8.693 |  | 0.003 |  | 0.023 |  |
| rs2746414 |  | 0.002 |  | 1 |  | 0.002 |  | 0.346 |  | 0.557 |  | 9.181e -4 |  |
| group * rs2746414 |  | 4.943e -4 |  | 1 |  | 4.943e -4 |  | 0.081 |  | 0.776 |  | 2.159e -4 |  |
| Residuals |  | 2.289 |  | 376 |  | 0.006 |  |  |  |  |  |  |  |

**Table S12 Descriptives regarding rs2746414 for serum C4 protein levels (g/L) between first-episode psychosis and healthy control**

| **group** | | **rs2746414** | | **Mean** | | **SD** | | **N** | |
| --- | --- | --- | --- | --- | --- | --- | --- | --- | --- |
| FEP |  | AG |  | 0.283 |  | 0.073 |  | 29 |  |
|  |  | GG |  | 0.280 |  | 0.082 |  | 152 |  |
| HC |  | AG |  | 0.255 |  | 0.071 |  | 35 |  |
|  |  | GG |  | 0.245 |  | 0.076 |  | 164 |  |

FEP: first-episode psychosis; HC: healthy control.
